# High-performance combinatorial optimization based on classical mechanics

**DOI:** 10.1126/sciadv.abe7953

**Published:** 2021-02-03

**Authors:** Hayato Goto, Kotaro Endo, Masaru Suzuki, Yoshisato Sakai, Taro Kanao, Yohei Hamakawa, Ryo Hidaka, Masaya Yamasaki, Kosuke Tatsumura

**Affiliations:** 1 Corporate Research and Development Center, Toshiba Corporation, 1 Komukai Toshiba-cho, Saiwai-ku, Kawasaki 212-8582, Japan.; 2 Software Systems Research and Development Center, Toshiba Digital Solutions Corporation, 72-34 Horikawa-cho, Saiwai-ku, Kawasaki 212-8585, Japan.; 3 ICT Solutions Division, Toshiba Digital Solutions Corporation, 72-34 Horikawa-cho, Saiwai-ku, Kawasaki 212-8585, Japan.

## Abstract

Quickly obtaining optimal solutions of combinatorial optimization problems has tremendous value but is extremely difficult. Thus, various kinds of machines specially designed for combinatorial optimization have recently been proposed and developed. Toward the realization of higher-performance machines, here, we propose an algorithm based on classical mechanics, which is obtained by modifying a previously proposed algorithm called simulated bifurcation. Our proposed algorithm allows us to achieve not only high speed by parallel computing but also high solution accuracy for problems with up to one million binary variables. Benchmarking shows that our machine based on the algorithm achieves high performance compared to recently developed machines, including a quantum annealer using a superconducting circuit, a coherent Ising machine using a laser, and digital processors based on various algorithms. Thus, high-performance combinatorial optimization is realized by massively parallel implementations of the proposed algorithm based on classical mechanics.

## Teaser

Massively parallel implementations of algorithms based on classical mechanics realize high-performance combinatorial optimization.

## INTRODUCTION

Combinatorial optimization problems appear in various social and industrial situations, so quickly solving such problems makes the society and industry more efficient. However, these problems are notoriously hard due to combinatorial explosion, an exponential increase in the number of candidate solutions depending on the problem size (*1*). Thus, novel computational approaches to combinatorial optimization have been expected. A well-known example is a quantum annealer (QA), which is based on quantum annealing (*2*–*4*) and its superconducting circuit implementation (*5*, *6*). The QA is an Ising machine designed to find ground states of Ising spin models (*7*). Such Ising machines are believed to be broadly useful, because the Ising problem belongs to the nondeterministic polynomial time (NP)–complete class (*7*), and consequently, many combinatorial optimization problems can be reduced to the Ising problem (*8*). Various Ising machines other than QA have been developed, including coherent Ising machines (CIMs) implemented with pulse lasers (*9*–*14*) and other kinds of optical Ising machine (*15*–*18*), as well as digital processors based on simulated annealing (SA) (*19*–*23*), which is a standard heuristic algorithm for combinatorial optimization (*1*, *24*, *25*), or other recently proposed algorithms (*26*–*29*).

A heuristic algorithm called simulated bifurcation (SB) has recently been proposed for accelerating combinatorial optimization (*30*). SB is a purely quantum-inspired algorithm, that is, it was derived from a classical-mechanical model corresponding to a quantum computer called a quantum bifurcation machine (QbM) (*30*–*33*), which is based on quantum adiabatic optimization using nonlinear oscillators exhibiting quantum-mechanical bifurcation phenomena. Consequently, SB is based on numerical simulation of adiabatic evolutions in classical nonlinear Hamiltonian systems exhibiting bifurcations (*34*). Different dynamical approaches have also recently been proposed (*35*–*41*). SB is not based on the gradient method, unlike other dynamical approaches such as the Hopfield-Tank model (*42*), simulated CIM (SimCIM) (*35*, *39*), and their variants, but based on adiabatic evolutions of energy conservative systems like purely adiabatic QA and QbM. [Such interesting contrast between QbM and CIM has been summarized in a review paper on this topic (*33*).]

Unlike SA in general cases, SB allows simultaneous updating of variables and therefore can easily accelerate combinatorial optimization through massively parallel processing using modern many-core processors such as field-programmable gate arrays (FPGAs) (*30*, *43*, *44*) and graphic processing units (GPUs) (*30*). An SB-based machine (SBM) implemented with a single FPGA, where about 8000 operations are performed in parallel, was able to find good approximate solutions of a 2000-spin Ising problem in 0.5 ms, about 10 times faster than a CIM (*30*). This result suggests that parallelizability is a key property of optimization algorithms for their acceleration by fully exploiting modern high-performance computing systems. In this direction of research, other parallelizable algorithms have also recently been proposed by mapping a given problem to a bipartite one and applying parallel SA updating to each group of spins (*26*–*28*). These new algorithms essentially rely on the same mechanism, although they are given different names: momentum annealing (MA) (*26*), stochastic cellular automata annealing (SCA) (*27*), and restricted Boltzmann machine (RBM)’s parallel stochastic sampling (*28*).

The previous results on SB demonstrate that SB is useful for quickly finding good approximate solutions. However, it remains unclear whether SB can find optimal solutions of large-scale problems. For enhancing the power of SB in terms of solution accuracy, in this work, we introduce two SB variants, named ballistic SB (bSB) and discrete SB (dSB), in addition to the original adiabatic SB (aSB). We solve various problems to compare the performance of bSB and dSB with that of aSB and other recently developed machines, including a QA, a CIM, and digital processors based on various algorithms. This benchmarking shows that bSB and dSB provide faster and more accurate optimizations than does aSB and that our new SBMs achieve high performance compared to the other machines. dSB can find optimal or near-optimal solutions of problems with up to one million spins, which aSB and bSB cannot achieve. Thus, high-performance machines for combinatorial optimization are realized by massively parallel implementations of the proposed algorithms based on classical mechanics.

## RESULTS

### bSB and dSB algorithms

The Ising problem is to find a spin configuration minimizing the Ising energy, defined as

$$E_{\text{Ising}}=-\frac{1}{2}\sum_{i=1}^{N}\sum_{j=1}^{N}J_{i,j}s_{i}s_{j}-\sum_{i=1}^{N}h_{i}s_{i}$$ (1)

where *s_i_* denotes the *i*th spin taking 1 or −1, *N* is the number of spins, *J*_*i*,*j*_ is the coupling coefficient between the *i*th and *j*th spins (*J*_*i*,*j*_ = *J*_*j*,*i*_ and *J*_*i*,*i*_ = 0), and *h_i_* is the local field on the *i*th spin. Since introducing an ancillary spin reduces the Ising problem to the one without local fields (see section S1), here, we focus on the Ising problem with no local fields (*h_i_* = 0).

To solve the Ising problem, QbM uses quantum-mechanical nonlinear oscillators called Kerr-nonlinear parametric oscillators (KPOs), each of which can generate a Schrödinger cat state, i.e., a quantum superposition of two oscillating states, via a quantum-mechanical bifurcation (*31*). Such a KPO has recently been realized experimentally using superconducting circuits (*45*, *46*). However, the realization of a large-scale QbM will require a long time. On the other hand, it was found that a classical-mechanical model corresponding to QbM, referred to as a classical bifurcation machine (CbM), also works as an Ising machine (*31*, *33*). In this case, we can efficiently simulate such a classical machine using present digital computers, instead of building real machines. [This is not the case for QbM, because QbM can also be used as a universal quantum computer (*47*, *48*), which is classically unsimulatable.] This simulation approach paves the way for large-scale Ising machines with all-to-all connectivity.

By modifying the equations of motion for CbM such that computational costs are reduced and the symplectic Euler method (*49*) is applicable, we obtain the following Hamiltonian equations of motion for aSB (*30*)

$${\overset{\cdot }{x}}_{i}=\frac{\partial H_{\text{aSB}}}{\partial y_{i}}=a_{0}y_{i}$$ (2)

$${\overset{\cdot }{y}}_{i}=-\frac{\partial H_{\text{aSB}}}{\partial x_{i}}=-[x_{i}^{2}+a_{0}-a(t)]x_{i}+c_{0}\sum_{j=1}^{N}J_{i,j}x_{j}$$ (3)

$$H_{\text{aSB}}=\frac{a_{0}}{2}\sum_{i=1}^{N}y_{i}^{2}+V_{\text{aSB}}$$ (4)

$$V_{\text{aSB}}=\sum_{i=1}^{N}(\frac{x_{i}^{4}}{4}+\frac{a_{0}-a(t)}{2}x_{i}^{2})-\frac{c_{0}}{2}\sum_{i=1}^{N}\sum_{j=1}^{N}J_{i,j}x_{i}x_{j}$$ (5)

where *x_i_* and *y_i_* are, respectively, the position and momentum of a particle corresponding to the *i*th spin, dots denote time derivatives, *a*(*t*) is a control parameter increased from zero, *a*_0_ and *c*_0_ are positive constants, and *V*_aSB_ is the potential energy in aSB.

**Fig. 1:**
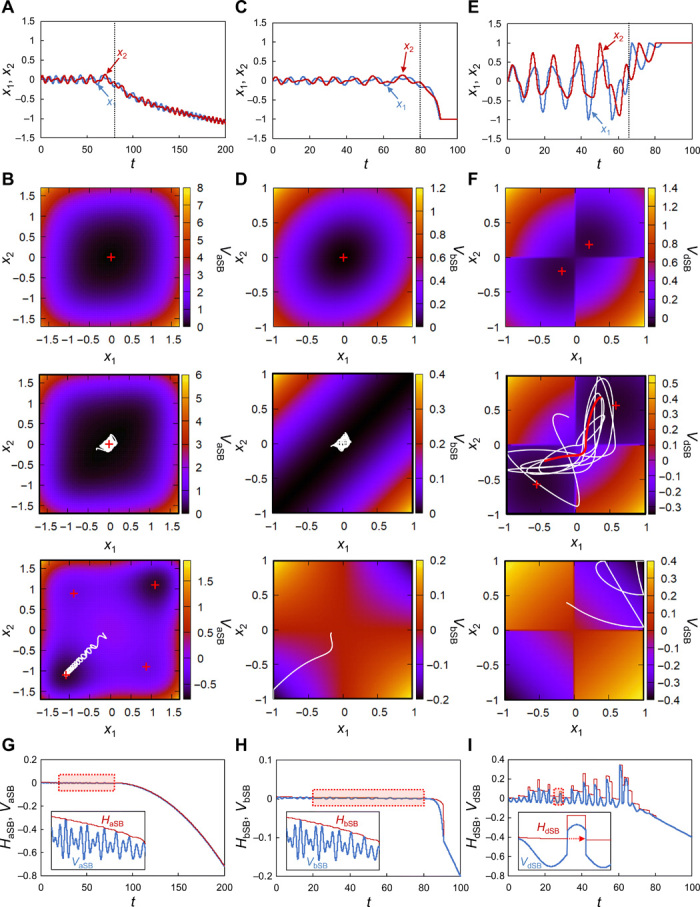
Dynamics in three SB algorithms. Two-spin ferromagnetic Ising problem (*J*_1,2_ = *J*_2,1_ = 1) is solved by aSB, bSB, and dSB with *a*(*t*) = 0.01*t*, *a*_0_ = 1, and *c*_0_ = 0.2. (**A**) Time evolutions of *x*_1_ and *x*_2_ in aSB. (**B**) Potential energies *V*_aSB_ in aSB at the initial (top), intermediate (middle), and final (bottom) times. The vertical dotted line in (A) indicates the intermediate time. Curves in white are trajectories between the initial and intermediate times (middle) and between the intermediate and final times (bottom). Local minima of *V*_aSB_ are shown by + in red. (**C** to **F**) Corresponding results for bSB (C and D) and dSB (E and F). (**G** to **I**) Time dependences of potential energy and total energy in aSB (G), bSB (H), and dSB (I). In each panel, the inset magnifies the part in the red dashed square. The dashed arrow in the inset in (I) indicates tunneling-like behavior in dSB. The red bold curve in the middle of (F) shows the trajectory corresponding to this inset.

To qualitatively explain the operation principle of aSB, we show an example of the dynamics in aSB in Fig. 1 (A and B), where the ferromagnetic two-spin Ising problem (*J*_1,2_ = *J*_2,1_ = 1) with solutions *s*_1_ = *s*_2_ = ± 1 is solved as the simplest problem. All positions and momenta are randomly set around zero at the initial time. The initial potential has a single local minimum at the origin (top and middle of Fig. 1B), so the particles circulate around the origin (Fig. 1A and middle of Fig. 1B). When *a*(*t*) becomes sufficiently large, bifurcations occur, that is, multiple local minima of the potential appear. Then, the particles adiabatically follow one of the minima. Consequently, each *x_i_* bifurcates to a positive or negative value (Fig. 1A and bottom of Fig. 1B). Since two local minima corresponding to the two solutions have lower energies and appear earlier than the other two local minima, the particles successfully find one of the solutions. Last, the sign of *x_i_*, sgn(*x_i_*), gives the *i*th spin, *s_i_*, for the solution of the Ising problem. It has been empirically found that aSB works well for much larger-scale and more complex problems (*30*).

This aSB relies on the fact that the second term in *V*_aSB_ is approximately proportional to the Ising energy at the final time (*30*). In this approximation, analog errors arise from the use of continuous variables (positions) instead of discrete variables (spins). These analog errors in aSB may degrade solution accuracy and result in approximate solutions. Such analog errors in different dynamical approaches have also been discussed (*38*, *40*).

To suppress analog errors, we introduce perfectly inelastic walls at *x_i_* = ± 1. That is, at each time, we replace *x_i_* with its sign, sgn(*x_i_*) = ± 1, and set *y_i_* = 0 if ∣*x_i_*∣ > 1. These walls force positions to be exactly equal to 1 or −1 when *a*(*t*) becomes sufficiently large. Moreover, we drop the fourth-order terms in *V*_aSB_, because the inelastic walls can play a role similar to the nonlinear potential walls. We thus obtain the following equations

$${\overset{\cdot }{x}}_{i}=\frac{\partial H_{\text{bSB}}}{\partial y_{i}}=a_{0}y_{i}$$ (6)

$${\overset{\cdot }{y}}_{i}=-\frac{\partial H_{\text{bSB}}}{\partial x_{i}}=-[a_{0}-a(t)]x_{i}+c_{0}\sum_{j=1}^{N}J_{i,j}x_{j}$$ (7)

$$H_{\text{bSB}}=\frac{a_{0}}{2}\sum_{i=1}^{N}y_{i}^{2}+V_{\text{bSB}}$$ (8)

$$V_{\text{bSB}}=\frac{a_{0}-a(t)}{2}\sum_{i=1}^{N}x_{i}^{2}-\frac{c_{0}}{2}\sum_{i=1}^{N}\sum_{j=1}^{N}J_{i,j}x_{i}x_{j}\;\begin{array}{c} \text{when}\;|x_{i}|\leq 1\;\text{for all}\;x_{i}\\ \text{otherwise}\;V_{\text{bSB}}=\infty \end{array}$$ (9)

This artificial dynamical system is the basis of the bSB. In bSB, we solve Eqs. 6 and 7 by the symplectic Euler method, where time is discretized with a time step Δ*_t_*, together with the updating for the inelastic walls (see Methods for a detailed algorithm). (If we solve these equations by the standard Euler method, instead of the symplectic Euler method, then solution accuracy becomes lower. See section S2.)

Similar modification to the above walls has been proposed for SimCIM (*39*), but this algorithm is based on the gradient method, like the Hopfield-Tank model (*42*), and also uses stochastic processes. In contrast, bSB is based on a classical-mechanical system conserving energy except for the inelastic walls and adiabatic changes of energy and also uses deterministic processes except for initial value setting. As a result, the performance of bSB is quite different from that of SimCIM (see section S3 for the comparison between bSB and SimCIM).

In bSB, it is sufficient to increase *a*(*t*) to *a*_0_. Then, the final potential has only the second term related to the Ising energy. Consequently, the following condition is satisfied for all *i* at the final time (see section S4)

$$\Delta E_{i}=2s_{i}\sum_{j=1}^{N}J_{i,j}s_{j}\geq 0$$ (10)

where *s_i_* = sgn (*x_i_*) is the sign of *x_i_* and Δ*E_i_* represents the change in the Ising energy for a flip of *s_i_*. Note that Eq. 10 is a sufficient condition to show that the spin configuration is a local minimum of the Ising problem. Hence, solutions obtained by bSB are at least local minima in the Ising problem. In contrast, this is not necessarily guaranteed in aSB because of its nonlinear potential terms. (This means that solutions obtained by aSB can sometimes be improved by a naïve local search based on sequential spin flips.) This is another reason why bSB should achieve higher accuracy than aSB. Throughout this work, we linearly increase *a*(*t*) from 0 to *a*_0_ and set *a*_0_ to 1.

Here, we show an example of the bSB dynamics using the same two-spin problem as above. The initial potential has a single local minimum at the origin (top of Fig. 1D) and particles circulate around the origin (Fig. 1C and middle of Fig. 1D), as in aSB. In bSB, however, stable points suddenly jump from the origin to the walls at *x_i_* = ± 1, which prevents adiabatic evolution. Instead, particles move toward walls in a ballistic manner (Fig. 1C and bottom of Fig. 1D). This ballistic (nonadiabatic) behavior in bSB leads to fast convergence to a local minimum of *V*_bSB_ and, consequently, to fast optimization.

For further improvement, we introduce another variant of SB by discretizing *x_j_* to sgn(*x_j_*) in the second term in Eq. 7

$${\overset{\cdot }{x}}_{i}=\frac{\partial H_{\text{dSB}}}{\partial y_{i}}=a_{0}y_{i}$$ (11)

$${\overset{\cdot }{y}}_{i}=-\frac{\partial H_{\text{dSB}}}{\partial x_{i}}=-[a_{0}-a(t)]x_{i}+c_{0}\sum_{j=1}^{N}J_{i,j}\text{sgn}(x_{j})$$ (12)

$$H_{\text{dSB}}=\frac{a_{0}}{2}\sum_{i=1}^{N}y_{i}^{2}+V_{\text{dSB}}$$ (13)

$$V_{\text{dSB}}=\frac{a_{0}-a(t)}{2}\sum_{i=1}^{N}x_{i}^{2}-c_{0}\sum_{i=1}^{N}\sum_{j=1}^{N}J_{i,j}x_{i}\text{sgn}(x_{j})\;\begin{array}{l} \text{when}\;|x_{i}|\leq 1\;\text{for all}\;x_{i}\\ \text{otherwise}\;V_{\text{dSB}}=\infty \end{array}$$ (14)

The discrete version of bSB, namely, dSB (see Methods for a detailed algorithm), is expected to achieve higher accuracy than that of bSB, because analog errors are further suppressed by discretization. We found that this discretization is effective for bSB but not for other algorithms such as SimCIM and aSB (see sections S3 and S5).

Note that the singularity on the boundaries between positive and negative regions has been intentionally neglected. This leads to a violation of conservation of energy across boundaries and, hence, to escape from local minima over potential barriers, as shown below. In this sense, dSB goes beyond naïve algorithms based on classical-mechanical systems conserving energy (except adiabatic change), such as aSB and bSB. We also increase *a*(*t*) to *a*_0_ for the convergence to a local minimum of the Ising problem at the final time, as in bSB (see section S4).

Figure 1 (E and F) shows an example of the dSB dynamics using the same two-spin problem as above. Unlike aSB and bSB, the particles go back and forth between two local minima through the potential barriers (Fig. 1E and middle of Fig. 1F). This is similar to quantum tunneling, as depicted by the inset in Fig. 1I. This tunneling-like behavior is possible due to the above-mentioned neglect of the singularity on the boundaries; otherwise, the potential walls on the boundaries prevent this tunneling. In contrast, conservation of energy prevents such tunneling in aSB and bSB, as suggested by the insets in Fig. 1 (G and H). Thus, it is expected that this tunneling-like behavior will help dSB to escape local minima of the potential, and hence, dSB will outperform aSB and bSB in terms of solution accuracy.

Note that both bSB and dSB maintain the advantage of aSB over SA, namely, high parallelizability. Therefore, they are expected to realize both high speed and high accuracy simultaneously.

### Performance for a 2000-spin Ising problem with all-to-all connectivity

To compare the performance of bSB and dSB with that of aSB, we solved a 2000-spin Ising problem with all-to-all connectivity. This problem was named K_2000_ and previously solved by aSB (*30*), a CIM (*11*), and a recently developed digital chip called STATICA (*27*), which is based on the above-mentioned SCA. This problem can be regarded as a 2000-node MAX-CUT problem (*11*, *30*); so, here, we evaluate performance using cut values (see section S6 for the definition of the cut value and the relation between MAX-CUT and the Ising problem). The best cut value for K_2000_ is estimated to be 33,337 (see section S7).

**Fig. 2:**
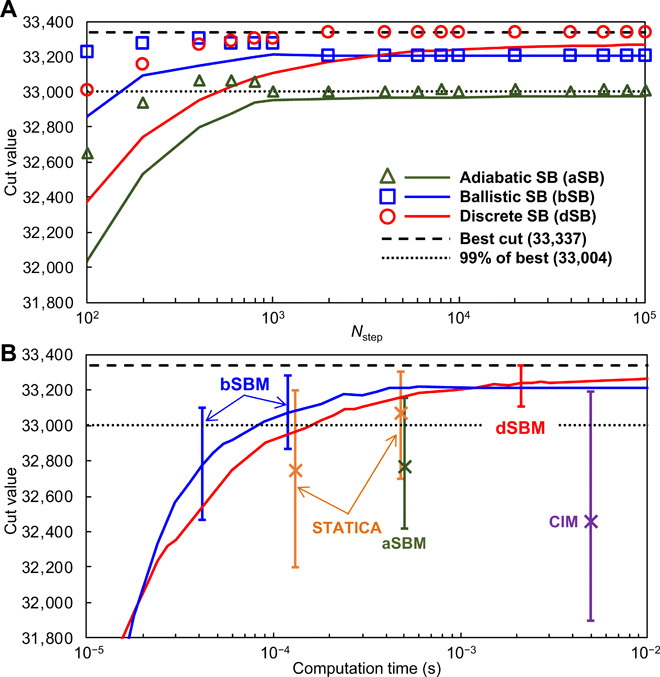
Comparison of performance for a 2000-spin Ising problem with all-to-all connectivity (K_2000_). (**A**) Average (lines) and maximum (symbols) cut values for 1000 trials. *N*_step_ is the number of time steps. The time step Δ*_t_* is set to 0.5 (aSB) or 1 (bSB and dSB). (See Methods for other settings.) (**B**) Computation times for our bSB and dSB machines (bSBM and dSBM) implemented with single FPGAs and three previously developed machines: CIM (*11*), aSB machine (aSBM) (*30*), and STATICA (*27*). (The results by STATICA are predicted ones by a simulator. The other results are measured ones using real machines.) The cut values are final ones in each trial, where the computation times of bSBM and dSBM are controlled by *N*_step_. Lines show average cut values for bSBM (blue) and dSBM (red). Crosses show average cut values for the other three machines. Bars show maximum and minimum cut values. The average, maximum, and minimum values are evaluated by 100 trials. The time step Δ*_t_* is set to 1.25 for both the bSBM and dSBM. (See Methods for other settings.)

The lines and symbols in Fig. 2A show average and maximum cut values, respectively, for 1000 trials as functions of the number of time steps, *N*_step_. (Throughout the paper, *N*_step_ denotes the total number of time steps for each trial and the values of cost functions (cut values or Ising energies) as functions of *N*_step_ are final values, not intermediate values, in each trail.) The results clearly show that both bSB and dSB outperform aSB in terms of both speed and accuracy. In addition, only dSB obtained the best value. On the other hand, best values obtained by bSB and aSB become lower for larger *N*_step_. This result suggests that the best values may be obtained accidentally by nonadiabatic processes in bSB and aSB. For large *N*_step_, dynamics becomes more adiabatic and the chance to obtain better solutions by nonadiabatic processes may be lost.

We implemented 2048-spin-size bSB and dSB machines (bSBM and dSBM) using single FPGAs (see section S8 for details) and solved K_2000_ by them. Figure 2B shows the comparison between our machines and the above three other machines (*11*, *27*, *30*), where the lines and the crosses show the average values of our machines and the others, respectively, for 100 trials, and the bars show the maximum and minimum values among the 100 trials. (The bars for our machines are shown at only typical computation times.) Only our dSBM obtained the best value in a short time (2 ms), thereby simultaneously realizing both high speed and high accuracy. Also, our bSBM is remarkably fast, about three times faster than STATICA (*27*), the previously fastest machine for K_2000_. Note that the results by STATICA for K_2000_ are predicted values by a simulator, and a real STATICA chip is still 512-spin size (*27*). On the other hand, in this work, we have implemented faster real machines.

### Benchmarking using time-to-solution and time-to-target

To evaluate the computation speed more quantitatively, here, we introduce two metrics: time-to-solution (TTS) and time-to-target (TTT). TTS is a standard metric for evaluating Ising machine speeds (*14*, *23*, *28*, *29*), defined as the computation time for finding an optimal or best known value with 99% probability. TTT uses a target value, instead of an optimal value, as a good approximate solution. In this work, we define the target as 99% of the optimal or best known value. TTS and TTT are formulated as *T*_com_ log (1 − 0.99)/ log (1 − *P*_S_) (*14*, *23*), where *T*_com_ is the computation time per trial and *P*_S_ is the success probability for finding the optimal (TTS) or target (TTT) value. *P*_S_ is estimated from experimental results with many trials. When *P*_S_ > 0.99, TTS and TTT are defined as *T*_com_.

**Fig. 3:**
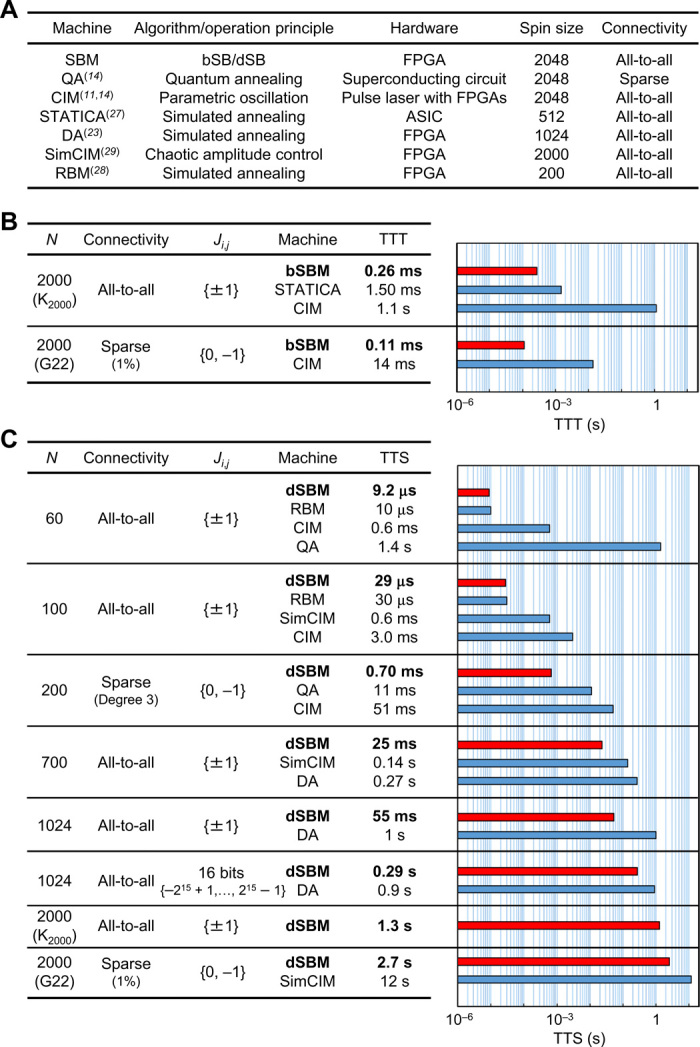
TTS and TTT benchmarking. (**A**) Machines compared in this benchmarking. Superscripts represent reference numbers for data sources. (**B**) Comparison of TTTs for two instances of 2000-spin size, K_2000_ and G22. (**C**) Comparison of TTSs. The values of the first six problems are medians for 100 (10 for CIM and 20 for QA) random instances. The bar graphs compare all TTTs and TTSs in log scale. For this benchmarking, the time step Δ*_t_* for bSBM and dSBM is set to the best value for each problem among five values (0.25, 0.5, 0.75, 1, and 1.25). [The same value of Δ*_t_* is used for 100 random instances of each of the first to sixth problems in (C).] The number of time steps, *N*_step_, is also optimized for TTT or TTS separately. See Methods and table S1 for the values of Δ*_t_* and *N*_step_ and other detailed information.

In the following, we compare TTS and TTT of our 2048-spin-size bSBM and dSBM with those of other recently developed machines shown in Fig. 3A. Since the bSBM can quickly find good approximate solutions and the dSBM can find optimal solutions of large-scale problems, we use the bSBM and dSBM for evaluations of TTT and TTS, respectively. TTS and TTT of other machines are cited or estimated from the data in the literature (see section S9 for details), because we could not use such machines for the present work. This limits the range of instances that can be used for this benchmarking. Also note that some machines are not the latest ones, as mentioned below.

Figure 3B shows the results of TTT. For K_2000_, the TTT of our bSBM (0.26 ms) is much shorter than those of STATICA (*27*) (1.50 ms, a predicted value by a simulator) and the CIM (*11*) (1.1 s). As a 2000-spin-size instance with sparse connectivity, we also solved G22, which is one of the well-known MAX-CUT benchmark instances called G-set and was solved by the CIM (*11*). For G22, the TTT of our bSBM is two orders of magnitude shorter than that of the CIM. These results demonstrate that our bSBM can find good approximate solutions faster than other recently developed machines of the same spin size. (TTTs of our machines for other G-set instances are provided in table S2.)

Next, we show the results of TTS in Fig. 3C. We start with the same two instances, namely, K_2000_ and G22. The TTS of our dSBM for them are 1.3 and 2.7 s, respectively. While TTS for K_2000_ has not been reported so far, TTS for G22 was evaluated with a SimCIM implemented on a FPGA (*29*), which is based on a recently proposed algorithm called chaotic amplitude control (CAC) (*29*, *40*). The TTS of the SimCIM is estimated at 12 s (see section S9). Thus, our dSBM has achieved a shorter TTS for G22 than that of the state-of-the-art machine. (TTSs of our machines for other G-set instances and the comparison between them and those of the SimCIM are provided in table S2 and fig. S6, respectively.)

We also solved other various instances of the Ising problem (MAX-CUT) by dSBM to compare it with other machines shown in Fig. 3A. For small-scale problems, we can simultaneously perform multiple trials using the 2048-spin-size machine by a block-diagonal structure of the *J* matrix, as done using a CIM (*14*). This so-called batch processing improves the success probability *P*_S_ by selecting the best result among multiple trials, while *T*_com_ is defined as the computation time per batch. In the limit as the number of trials per batch *N*_batch_ goes to infinity, *P*_S_ may exceed 0.99, and then TTS and TTT become *T*_com_ from the above definitions. In this sense, the TTS and TTT are well defined even for batch processing.

As shown in Fig. 3C, for two small-scale problems with 60 spins (all-to-all connectivity) and 200 spins (sparse connectivity), our dSBM achieved much shorter TTSs than those of a QA and a CIM (*14*). (This QA is not the latest version.) For 100-spin and 700-spin problems with all-to-all connectivity, the TTSs of the dSBM are much shorter than those of the SimCIM with CAC (*29*). These short TTSs of dSBM compared to the SimCIM come not from computation speed or implementations but the algorithmic advantage of dSB over CAC. That is, dSB needs fewer matrix-vector multiplications (MVMs), which is the most computation-intensive part in both algorithms, to obtain solutions than does the SimCIM with CAC. [The numbers of MVMs to solutions of the 100-spin and 700-spin problems are 9.4 × 10 and 8.1 × 10^4^, respectively, for dSB but 5.6 × 10^3^ and 7.8 × 10^5^, respectively, for CAC (*29*).] For two 1024-spin problems (all-to-all connectivity) with different ranges of *J*_*i*,*j*_, the dSBM achieved shorter TTSs than those of a Digital Annealer (DA) (*23*), which is based on an FPGA implementation of “Digital Annealer’s algorithm” developed from SA and outperformed CPU implementations of SA (*25*) and parallel tempering (*50*). (This DA is not the latest version.) Last, for 60-spin and 100-spin problems with all-to-all connectivity, the TTSs of the dSBM are comparable to those of another state-of-the-art machine based on an FPGA implementation of the above-mentioned RBM’s stochastic sampling (*28*), the size of which, however, is limited to 200 spins. Overall, we conclude that our bSBM and dSBM have achieved remarkably high performance for the present benchmark instances compared to the recently developed machines.

### Performance for ultralarge-scale Ising problems

**Fig. 4:**
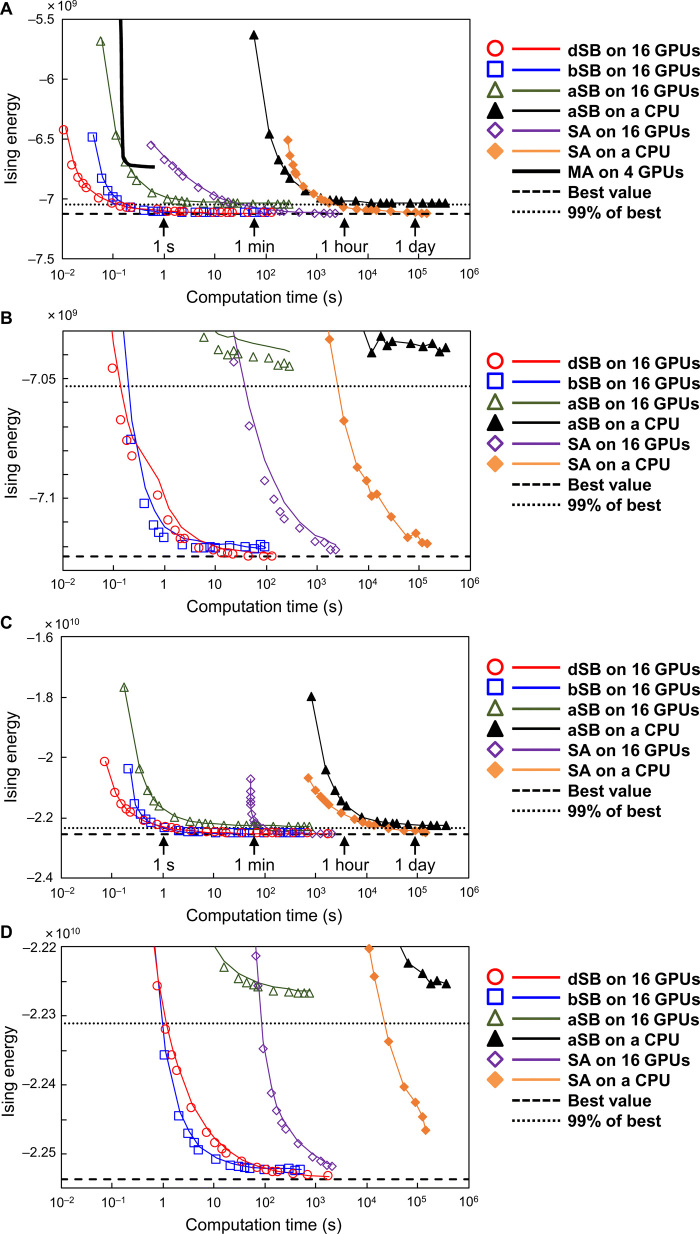
Results for ultralarge-scale Ising problems. (**A**) Computation times of aSB, bSB, dSB, and our best SA implemented on a 16-GPU cluster, aSB and SA (*25*) running on a CPU core, and MA implemented on a four-GPU cluster (*26*) for a 100,000-spin Ising problem with all-to-all connectivity. Each coupling coefficient *J_i,j_* was a randomly chosen 10-bit integer in {−2^9^ + 1,…,2^9^ – 1} (*26*). Lines and symbols represent average and best values, respectively, for 100 trials (one trial for aSB and SA running on a CPU). (**B**) Magnification of (A) around the estimated optimal value. (**C** and **D**) Corresponding results for a 1,000,000-spin Ising problem with a sparse connectivity of 1%. Each 1% nonzero *J*_*i*,*j*_ was a randomly chosen 10-bit integer in {−2^9^ + 1,…,2^9^ – 1}.

Last, we present the results for two ultralarge-scale Ising problems: a 100,000-spin problem with all-to-all connectivity and a 1,000,000-spin problem with a sparse connectivity of 1% (see section S7 for their detailed definitions for reproduction). Using a GPU cluster with 16 GPUs, we solved these by aSB, bSB, dSB, and our best implementation of SA (see section S10 for details). For comparison, we also solved them by aSB and SA (*25*) running on a CPU core. Figure 4 shows the results, where the result obtained by the four-GPU implementation of the above-mentioned MA (*26*) is also shown. The horizontal lines show optimal (dashed) and target (dotted) values estimated using a formula based on statistical mechanics (see section S7 for details) (*51*, *52*).

Figure 4A shows that all three GPU-cluster SBMs outperformed the MA machine (*26*) in terms of both speed and accuracy. Figure 4 (A and B) also show that the GPU-cluster implementation achieved about 1000 times speedup over a CPU core for aSB but only about 100 times for SA. This difference comes from the higher parallelizability of aSB than that of SA. The GPU-cluster bSBM and dSBM are faster than the GPU-cluster aSBM, because of their algorithmic advantage. Figure 4B shows that the dSBM achieved the closest value to the estimated optimal value, giving it the highest accuracy. As shown in Fig. 4 (C and D), similar results also hold for the 1,000,000-spin Ising problem with sparse connectivity.

## DISCUSSION

In this work, we have proposed two new variants of the SB algorithm, named bSB and dSB, both of which outperform the original aSB in terms of both speed and solution accuracy. dSB allows us to find optimal solutions of large-scale problems, which aSB and bSB cannot achieve. We have implemented 2048-spin-size bSBM and dSBM using single FPGAs. Our benchmarking with TTS and TTT has shown that the bSBM and dSBM can achieve remarkably high performance compared to other recently developed machines. GPU-cluster implementations of bSB and dSB also allow us to find nearly optimal solutions of ultralarge-scale problems with up to one million spins.

Last, we discuss possible future works on SB. First, it is important to check the performance of SB for a broader range of instances than those evaluated in this work, which were chosen to compare our machines with previously developed machines reported in the literature. It is known that a single solver cannot achieve the highest performance for all kinds of instances (*53*). Thus, we should examine what kinds of instances can be solved well by SB. Second, it is desirable to develop a technique for auto-tuning of parameters in SB, such as a constant *c*_0_ and a time step Δ*_t_*. In this work, we used the definition of *c*_0_ based on random matrix theory (*30*) and also chose the best value of Δ*_t_* among five values (see Methods for details). Such preliminary search for the best parameter values is often used for benchmarking to evaluate potential performance of solvers. In practical applications, however, it is desirable to determine appropriate parameter values automatically without preliminary search. Last, development of other variants of SB is an interesting possibility. In this work, we have focused on the Ising problem (MAX-CUT). The generalization of SB to broader classes of problem, e.g., combinatorial optimization with higher-order polynomial cost functions, is an interesting next target.

## METHODS

### Ballistic simulated bifurcation

In bSB, we numerically solve the Hamiltonian equations of motion given by Eqs. 6 and 7 using the symplectic Euler method (*49*), as in aSB (*30*). The updating rule for bSB is as follows

$$y_{i}(t_{k+1})=y_{i}(t_{k})+\{-[a_{0}-a(t_{k})]x_{i}(t_{k})+c_{0}\sum_{j=1}^{N}J_{i,j}x_{j}(t_{k})\}\Delta_{t}$$ (15)

$$x_{i}(t_{k+1})=x_{i}(t_{k})+a_{0}y_{i}(t_{k+1})\Delta_{t}$$ (16)

Here, Δ*_t_* is the time step and *t_k_* is the discretized time satisfying *t*_0_ = 0 and *t*_*k* + 1_ = *t_k_* + Δ*_t_*. After updating *x_i_*, we check whether ∣*x_i_*∣ > 1. If so, we replace *x_i_* with sgn(*x_i_*) and set *y_i_* = 0, which implements perfectly inelastic walls at *x_i_* = ± 1.

### Discrete simulated bifurcation

In dSB, we numerically solve Eqs. 11 and 12. The updating rule for dSB is as follows

$$y_{i}(t_{k+1})=y_{i}(t_{k})+\{-[a_{0}-a(t_{k})]x_{i}(t_{k})+c_{0}\sum_{j=1}^{N}J_{i,j}\text{sgn}[x_{j}(t_{k})]\}\Delta_{t}$$ (17)

$$x_{i}(t_{k+1})=x_{i}(t_{k})+a_{0}y_{i}(t_{k+1})\Delta_{t}$$ (18)

As in bSB, we replace *x_i_* with sgn(*x_i_*) and set *y_i_* = 0 if ∣*x_i_*∣ > 1.

### Efficient implementations of bSB and dSB

The bSB and dSB can be implemented more efficiently as follows. [A similar speedup technique has been used for SA (*25*).]

Storing $z_{i}(k)=\sum_{j=1}^{N}J_{i,j}x_{j}(t_{k})$, we can rewrite Eq. 15 as

$$y_{i}(t_{k+1})=y_{i}(t_{k})+\{-[a_{0}-a(t_{k})]x_{i}(t_{k})+c_{0}z_{i}(k)\}\Delta_{t}$$ (19)

$$z_{i}(k)=z_{i}(k-1)+\sum_{j=1}^{N}J_{i,j}\Delta x_{j}(k)$$ (20)

where Δ*x_j_*(*k*) = *x_j_*(*t_k_*) − *x_j_*(t_*k* − 1_). Note that Δ*x_j_*(*k*) often becomes zero around the final time. (This is not the case for aSB, making this implementation ineffective.) Hence, the product-sum operation in Eq. 20 can be accelerated by neglecting the terms corresponding to Δ*x_j_*(*k*) = 0.

In the case of dSB, instead of Δ*x_j_*(*k*), we use Δ*s_j_*(*k*) = sgn [*x_j_*(*t_k_*)] − sgn [*x_j_*(t_*k* − 1_)] as

$$z_{i}(k)=z_{i}(k-1)+\sum_{j=1}^{N}J_{i,j}\Delta s_{j}(k)$$ (21)

Since Δ*s_j_*(*k*) becomes zero more often than Δ*x_j_*(*k*), this implementation is more effective for speedup in dSB than in bSB.

### Parameter setting

In the SB algorithms, the time step Δ*_t_* and the constants *a*_0_ and *c*_0_ are appropriately set in advance. In this work, we set the constants as *a*_0_ = 1 and $c_{0}=\frac{0.5}{〈J〉\sqrt{N}}$, where 〈*J*〉 is defined as $〈J〉=\sqrt{\frac{\sum_{i,j}J_{i,j}^{2}}{N(N-1)}}$. This definition of *c*_0_ is based on random matrix theory (*30*). Although this setting of *c*_0_ may not be optimal for some instances, this is enough to achieve high performance presented in this work. On the other hand, the setting of Δ*_t_* is more sensitive to performance. We therefore selected the best value among five values: 0.25, 0.5, 0.75, 1, and 1.25. In Figs. 2A and 4, Δ*_t_* is set to 0.5 (aSB) or 1 (bSB and dSB). In Fig. 2B, Δ*_t_* is set to 1.25 for the bSBM and dSBM. The Δ*_t_* values used in Fig. 3 are provided in table S1. As mentioned in Discussion, automatic setting of *c*_0_ and Δ*_t_* is an important issue but beyond the scope of the present work and so left for future work.

## SUPPLEMENTARY MATERIALS

Supplementary material for this article is available at http://advances.sciencemag.org/cgi/content/full/7/6/eabe7953/DC1
